# Exploring the Functioning of Online Self-Organizations during Public Health Emergencies: Patterns and Mechanism

**DOI:** 10.3390/ijerph20054012

**Published:** 2023-02-23

**Authors:** Jinghao Chen, Qianxi Liu, Xiaoyan Liu, Youfeng Wang, Huizi Nie, Xiankun Xie

**Affiliations:** 1 School of Public Policy and Management, Guangxi University, Nanning 530004, China; 2 School of Languages and Communication Studies, Beijing Jiaotong University, Beijing 100044, China

**Keywords:** emergency response, online self-organization, social media use, patterns and mechanism, machine learning, public health emergencies

## Abstract

With the increasing use of social media, online self-organized relief has become a crucial aspect of crisis management during public health emergencies, leading to the emergence of online self-organizations. This study employed the BERT model to classify the replies of Weibo users and used K-means clustering to summarize the patterns of self-organized groups and communities. We then combined the findings from pattern discovery and documents from online relief networks to analyze the core components and mechanisms of online self-organizations. Our findings indicate the following: (1) The composition of online self-organized groups follows Pareto’s law. (2) Online self-organized communities are mainly composed of sparse and small groups with loose connections, and bot accounts can automatically identify those in need and provide them with helpful information and resources. (3) The core components of the mechanism of online self-organized rescue groups include the initial gathering of groups, the formation of key groups, the generation of collective action, and the establishment of organizational norms. This study suggests that social media can establish an authentication mechanism for online self-organizations, and that authorities should encourage online interactive live streams about public health issues. However, it is important to note that self-organizations are not a panacea for all issues during public health emergencies.

## 1. Introduction

A self-organization is a fundamental organizational form that fills in gaps left by government actions [1]. It is a voluntary and self-managed social entity composed of individuals or teams that achieve specific goals through the internal forces generated by its members [2,3]. In contrast to traditional government-led civic engagement, activists in the public prefer to participate in loosely organized non-governmental networks in order to advance their demands on the policy agenda [4]. These features make self-organizations stand out in crisis management situations in particular, because they are more flexible in form and can respond rapidly to individuals’ diversified needs [5,6,7].

The prevalence of social media has created a noticeable breeding ground for the formation of self-organizations in emergencies. Social media matters in crisis management because it provides a vital platform for the exchange of information during emergencies, which is often known as risk communication [8,9,10]. The information flowing then directs mutual aid to where it is needed [11]. Real-time information sharing and interactions on social media can effectively reduce the imbalance of risk information and help stakeholders in crises to quickly understand their situation [12] Government departments can utilize social media as a tool for disseminating information about relief and crisis situations. Meanwhile, online users can use social media to make requests for assistance, offer help, and share knowledge [13]. Through online interaction and information exchange, individuals facing similar risks or who embrace the same goals begin to spontaneously construct an intangible network with shared interests, which is the embryonic form of the online self-organization.

The combination of social media and self-organization has come to play a vital role in the disasters. Realizing the potential benefits of online self-organizations, scholars have begun to pay attention to its effects during public health emergencies. For example, Hughes and Tapia (2015) found that Twitter was used shortly after the 2010 Haiti Earthquake to update statuses and was embedded in the rescue activities of self-organized volunteer teams [14]. Ntontis et al. (2022) noted that citizens increasingly turned to Facebook mutual aid groups to offer and request various types of practical, emotional, and informational support [15]. These findings indicate that when formal organizations are unable to perform their duties in a crisis individuals resort to social media and voluntarily form informal groups to offer mutual support [16,17].

Although the above studies have recognized the importance of self-organization in crisis management, they still regard self-organizations as a derivative phenomenon in the process of social media use, and did not examine this phenomenon further. However, scholars have emphasized the instrumental value of social media in relief activities, illustrating its function of disseminating information in a crisis [18,19,20,21,22]. Furthermore, they have suggested that stakeholders in both online and offline groups could use social media as a tool of information discovery when carrying out relief activities or fulfilling tasks that government or other formal groups are not able to [7,23,24,25]. However, online self-organizations should be recognized as independent organisms of social media and even of self-organization. Any form of organization sets its boundaries by identifying key goals, structures, functions, and working mechanisms [4]. As an informal organization, online self-organization becomes a tangible entity through the integration and cooperation of various online groups that all may share different goals and interests [26,27]. Existing studies on self-organizations and social media have focused only on social media as a tool for information communication while ignoring its socio-spatial attributes, which are the basis for the formation of self-organizations, namely user communication processes, interactive relationships, and self-organization formed upon these bases. This prevents current research from fully illustrating the overall picture of the self-organization process in social media.

Based on the above discussion, this paper strives to comprehensively describe the active growth and development of online self-organization. In doing so, this research sheds light on the functioning of online self-organizations in public emergency situations by exploring their patterns and mechanism. (1) Using Weibo help-seeking data during the Shanghai lockdown in 2022, this study utilizes machine learning and social network analysis to comprehensively characterize the high-frequency interaction behaviors of online users to track the micro-evolution of their self-organizing activities and summarize the patterns of online self-organizations. (2) Second, this study combines different sources of relief information: news reports, help websites, and group chat records to deconstruct the mechanism of the online self-organizations. (3) This study employs data-driven methods and applies algorithms and computational tools to process complex data on social media and mine users’ micro-behaviors and high-frequency interaction networks contained therein to fully reveal the essence of online self-organizations in public health emergencies.

## 2. Theory and Research Questions

### 2.1. Self-Organizations in Crisis Management

The emergence of a self-organization represents the transition of social systems from disorder to order, which is regularly observed during public emergencies [24,28]. According to Haken and Fraser (1989), a system is considered self-organized if it acquires a spatial, temporal, or functional structure without exterior interference [2]. Trust and civic volunteerism are social capitals that facilitate the development of informal organizations. Trust is the cornerstone of effective interaction within an organization, while civic volunteerism is the psychological driver of collective actions [22,29]. Reciprocity also plays a role, stimulating the ongoing exchange of resources and information between a self-organization and the external environment. When this stimulus disappears the self-organization may face disintegration [30]. In regards to the internal composition of self-organizations, all components work in parallel to deal with different needs, and are integrated through a shared commitment to common goals that define the organizational boundaries [28].

Self-organizations, also known in crisis management as “emergent groups”, arise from a specific goal that requires a rapid response, and will disintegrate when the goal is achieved [31]. In contrast to formal organizations, self-organizations pursue rapid action and goal orientation, and their flexible organizational design and duration can help them adapt to the complex circumstances of emergencies [28]. During a crisis, people will spontaneously contribute their time, knowledge, physical strength, and materials through official and informal channels to restore social order. In their study of volunteering in crisis management, Simsa et al. (2019) found that spontaneous voluntary activities partially replaced the official response system, which led to the self-organization phenomenon [26]. Tu (2022) stated that Chinese self-organizations were critical in delivering services to families in the most affected communities and regions during the start of the COVID-19 pandemic [3]. The more likely it was that officially-registered NGOs failed to deliver goods and supplies, the more remarkable the self-organizational efforts were.

### 2.2. Social Media Use in Crisis Management

Scholars have considered social media as an online community for risk communication, where people can not only upload crisis information, needs, and concerns, but can also access authoritative announcements from official accounts [32,33,34]. This suggests that disaster rescue organizations of all types can make use of social media to obtain crisis information, determine the current state of affairs, and provide people-centered rescue services. Risk communication is not a one-way dissemination of risk information but an interactive process in which information and opinions are exchanged between individuals, groups, and institutions and, in addition to risk information, related opinions and reactions should also be considered [35]. In contrast to traditional media, social media emphasizes equal dialogue and diverse participation of information topics, allowing anyone to post information as well as repost and comment on others’ posts [22]. In this regard, social media provides not only a medium for disseminating information about a crisis, but also a forum for public participation in online discussions [36,37].

Several studies have already confirmed the value of social media in crisis management (Table 1).

### 2.3. Online Self-Organizations: The Organisms of Social Media and Informal Groups

In contrast to traditional offline self-organization, online self-organization relies on social media for information communication, leading to a non-fixed and non-institutionalized organizational design [3]. This flat, decentralized network structure reduces barriers to information dissemination and can extensively exploit social media’s “small world” effect to coordinate rapid responses to crisis information [41]. In Weibo, for example, users can join or create topics using Tags, or engage in discussions on super-topic squares to express specific needs or concerns, leading to the aggregation of individuals’ attention and event information, creating associations between them as well as encouraging a continuous flow of users to form social networks [42]. Thus, when a crisis erupts, online users can discuss it online to mobilize public opinion and promote the coupling of problematic and political windows, or spontaneously form relief teams via the Internet to offer support to help-seekers [22]. The exchange of information on social media effectively directs the flow of people and resources, which contributes to the rapid restoration of order in times of crisis [43].

Social media facilitates the alignment of resource supply and service demand both within and outside self-organizations, promoting the formation of online self-organizations. From a demand perspective, self-organization is task-oriented, operating according to spontaneous orders [26], and first forms around specific goals and interests [27]. Scholars have argued that self-organizations emerge when public needs are not met due to market and government deficiencies [5,44]. For example, residents in crisis may come together to form networks or organizations to provide mutual aid and support [45]. On the supply side, self-organizations may continue to receive resources such as volunteer support, donations, and other forms of assistance throughout their development, and may adjust their goals in response to changes in the crisis situation [24]. Social media can serve as an online platform for connecting those in need with those able to provide assistance. During a crisis, the constant sharing of information on social media can help direct resources from the supply side to the demand side, facilitating the formation of informal, virtual, and non-institutionalized social assistance networks [25].

With the popularity of social platforms, online self-organization has become a vital rescue force during crises. Kaufhold and Reuter (2016) studied how social media is embedded in voluntary organizations during a crisis and found that Twitter is used primarily for information release, while Facebook is more used by the online community to carry out comprehensive volunteering activities [25]. Silver and Matthews (2017) interviewed residents of Ontario, Canada, and revealed that people used Facebook for help and disaster relief when tornadoes hit [31]. Whittaker et al. (2015) examined the role of informal volunteer groups in crisis management, noting that they can provide additional relief to offset the incompetence of formal organizations [7]. Zhao and Wu (2020) have suggested that the flexibility of self-organizations and the experience of its members fulfill people’s special needs in a crisis [1]. Tu (2022) found that self-organizations have been competent in mobilizing resources and engaging in cooperation for the prevention and control of COVID-19 in Wuhan, China [3].

Although researchers have noticed the use of social media in disaster rescue activities [46,47], previous studies have regarded online self-organizations as more of a derivative of social media applications, without comprehensively exploring their process of formation. At the same time, they have mainly used case studies and content analysis methods, and have not made full use of big data from social media. This has also restricted their overall grasp of the online self-organization operation process. However, social media records a large amount of data on organizational activities, providing valuable resources for exploring self-organizing processes for online relief during crises. By mining and analyzing these texts and data, we can better understand the nature of online self-organizations. Thus, the research questions of this study are as follows:

RQ1: What types of network entities participate in online self-organized rescue in public health emergencies?

RQ2: How many types of network patterns are there for entities participating in online self-organized rescue in public health emergencies?

RQ3: What is the evolution process of online self-organized rescue for public health emergencies?

RQ4: How are online self-organized rescue groups organized during public health emergencies, and how do they function during public health emergencies?

## 3. Methods

### 3.1. Analysis Framework

**Figure 1:**
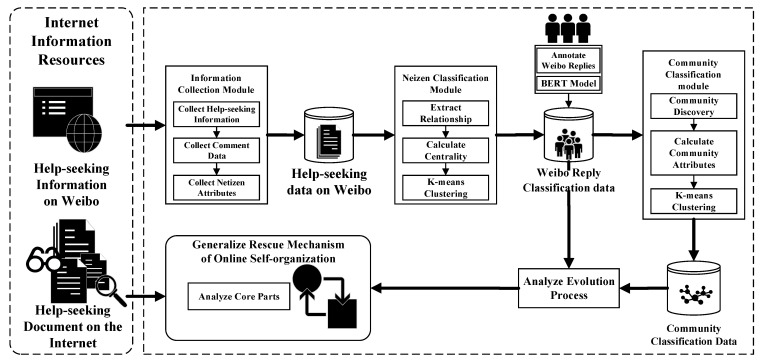
Research Framework.

The research framework consisted of five parts. The first part was the information collection module, which used web crawlers to collect online help information, related comments, and netizen attributes data to form an initial microblog help dataset. The second part was the netizen classification module, in which the network relationship data of netizens’ comments, forwarding, and replies were first extracted, then the Degree Centrality, Betweenness Centrality, Closeness Centrality, etc., were calculated and combined with netizen attribute data. K-means clustering was used to divide netizens into categories, revealing the composition of online help-seeking netizens. The team also labeled netizens’ behavior and used the BERT model (Bidirectional Encoder Representations from Transformers) to achieve automated classification. The third part was the community classification module, in which the community classification algorithm was used to detect the possible communities in the nodes, then the properties of each community (e.g., community size, member type, member behavior, graph density, cluster coefficient, and average path length) were calculated, after which K-means clustering was used to classify the communities and analyze and describe the characteristics of each category. The fourth part was the self-organizing evolution process explanation module, which was developed based on the analysis results and datasets generated by the first three modules, which revealed the macro-evolution process of online self-organizing assistance. The fifth part was the summary of the self-organizing assistance mechanism. The analysis materials used in this part came from two sources: the analysis results and datasets from the first four parts, and external information sources (e.g., news reports, organizational websites, aid materials, group chat records, etc.). By sorting out the core links of the self-organizing assistance mechanism, we ultimately revealed the systematic laws of online self-organization during unexpected public health emergencies (See Figure 1).

### 3.2. Data

The data used in this research came from the Sina Yuqingtong big data system (https://www.yqt365.com/ (accessed on 18 June 2022)), which has signed a data purchase agreement with Sina Weibo. Using the Sina public opinion database, which stores more than 60 million pieces of data from Weibo each day, we were able to access all data published by users on Sina Weibo during the past two years. The research population comprised a total 47,173 Sina Weibo users, and the user data were obtained by backtracking through their posts.

The specific data acquisition procedure was as follows. First, the research team collected posts of help-seeking information, mutual aid guides, and data on diagnosed COVID-19 cases uploaded to Weibo by Shanghai residents during the local COVID-19 lockdown period from the end of February 2022 to the beginning of June, 2022. Mutual aid guides referred to manuals compiled by volunteers and published on the Internet for self-rescue or assistance during the lockdown period, and included the Shanghai Epidemic Mutual Aid Guide and the Shanghai Epidemic Prevention Guide 4.0. These materials were mainly used to support the analysis of the online self-organized rescue mechanism. By setting the post location to Shanghai and the time range from 1 March to 31 May 2022, the lockdown period of Shanghai, we searched Weibo posts using keywords such as “Shanghai Mutual Aid”, “Anti-epidemic Mutual Aid”, and “Shanghai Epidemic Help”. We downloaded 26,106 help-seeking data from the platform, with 4893 Weibo posts containing replies to help-seeking information, by screening manually. Subsequently, our team used Python crawlers to capture data about user information, reposts, replies, and likes related to these posts. In the end, our data comprised a total 47,173 pieces of user data, 112,528 user reply data, and 75,666 relation pairs. Each piece of user data included the poster’s name, their number of followers, their number of posts, their reply content, and the reply time. Additionally, to examine the relationship between the evolution process of online self-organizations and changes occurring in the external environment, we also collected data on daily new diagnosed cases and new asymptomatic cases in Shanghai during the defined time period from the Shanghai Health Commission website.

### 3.3. Research Methods

This study was conducted from June to August in 2022, immediately after the termination of the COVID lockdown in Shanghai. The research team adopted a hybrid research method combining quantitative and qualitative approaches. The online self-organizing process produced a great deal of data, which provided a rich data source for quantitative research and helped us to explore and reveal comprehensive rescue patterns of the online self-organizations. We used the BERT algorithm to classify the massive number of replies we had collected to analyze netizens’ behaviors and utilized Gephi, social network analysis software, to determine potential online communities. We adopted K-means clustering to classify the patterns of the online groups and communities. All of these are forms of the quantitative method. At the same time, the operation mechanism of online self-organization is composed of numerous elements and links which manifested through the organizations’ daily management activities and needs, which were compiled and explained through observation, interview, induction, distillation, and abstraction. For this part of our study, it was more suitable to use qualitative analysis methods to conduct the interviews and analyze the relevant documents and materials. Thus, the combination of the two methods was best suited to provide an overview of the online self-organizing process. Specifically, the research methods used in this study are as follows:

1. Content analysis. We classified the replies in the comment area of original posts to determine repliers’ behavior. The results of this analysis served as a training and test set of the BERT model. Ultimately, we annotated 7000 data pieces in this round by employing the same annotation method as the first round of analysis and classified the replies into three types (see Table 2).

2. Text classification. As the number of replies was extremely large, making it difficult to manually annotate all the reply data, this study adopted the BERT model as developed by the Hugging Face community for natural language processing. The pretraining model was BERT-based-Chinese, and we used the fine-tuned data set which had been annotated in the second round of analysis, of which 70% was used as a training set and 30% was used as a test set. The iterative times of training model (Epoch) was 3, the Bath Size was 16, the Max Seq Length was 300, and the Learning Rate was 2 × 10^−5^. The accuracy rate, recall rate, and *F*-value of the final training model are shown in Table 3. The classification results of the model are shown in Table 4. The noresponse category refers to the nodes that were mentioned (@ in Weibo) but did not include a response, meaning the data contained no reply content.

3. Social network analysis. Gephi, a social network analysis and visualization software package, was employed to calculate the in-degree, out-degree, betweenness centrality, and closeness centrality of each node in the self-organized network, which can reflect the importance of each node in the network. Additionally, the nodes were divided into different communities using the Statistical Inference module of Gephi, which followed the Bayesian inference optimization criterion and used the same convergent heuristic algorithm as the Louvain algorithm. We also calculated the graph density, aggregation coefficient, and average path length for each community to reflect the closeness between nodes within the communities.

4. Clustering analysis. In clustering analysis, we employed the K-means clustering algorithm from the SKlearn machine learning library, a commonly used algorithm to mine homogeneous data and distinguish heterogeneous data. The algorithm first divided the data into K clusters and then calculated the Euclidean distance of each node to each cluster, and assigned it to the nearest cluster. The K-means clustering algorithm iterates until the criteria is reached (i.e., maximum sample similarity within clusters and minimum sample similarity between clusters).

## 4. Results

### 4.1. The Patterns and Evolution of Online Self-Organizations

#### 4.1.1. The Patterns of Online Self-Organized Groups

K-means clustering was employed to explore the structural characteristics and patterns of online self-organized groups. We selected six critical indicators as the clustering standards: number of followers, number of Weibo posts, in-degree, out-degree, betweenness centrality, and closeness centrality. Specifically, number of followers represented the influence of the node; number of Weibo posts represented the activity of the node; in-degree represented the number of times the node was mentioned by other nodes; out-degree represented the number of times the node referenced to or mentioned other nodes; betweenness centrality represented the number of shortest path through the node in the network, reflecting the node’s ability to control the information exchange in the network; finally, closeness centrality represented the average distance from one node to all other nodes in the network, which could range from 0 to 1, and the bigger its value, the farther its distance to other nodes. The parameter K of K-means clustering was determined by the elbow method. In the end, we divided the online self-organized groups into ten classes. Results are shown in Table 5.

**Figure 2:**
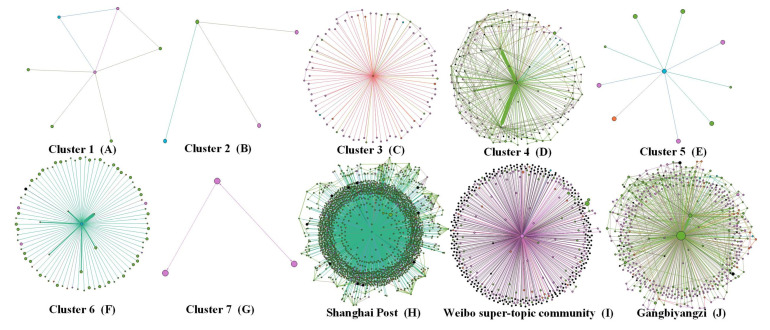
Patterns of Online Self-Organized Groups.

According to our clustering results, online self-organized groups contain seven patterns, with three outliers that do not belong to any pattern. Each pattern and outlier was characterized to reveal the composition of online self-organized groups. In Figure 2, the node depth of each node is 1.

Cluster 1 refers to Shanghai netizens active on Weibo. As shown in Table 5, this cluster has middle-range indicator values (i.e., mean of in-degree = 2.20, mean of out-degree = 1.22, mean of betweenness centrality = 29,038.48, mean of closeness centrality = 0.19). This group has a relatively high number of followers (1,622,873.46) and posts (43,643.85) among these seven categories. These Shanghai netizens have been active on Weibo for a long time. Their online behaviors manifest primarily in consulting relief measures, raising epidemic prevention problems, participating in epidemic discussions, and providing encouragement and advice. They attend small-scale online gatherings and are permanently active in various local topics on Weibo (see Figure 2A).

Cluster 2 contains Shanghai netizens who participate in discussions temporarily. As seen in Table 5, of the seven groups, the Cluster 2 group has relatively few followers (9811.44) and posts (1782.62). Furthermore, it has the highest mean of closeness centrality (0.81), the lowest mean of in-degree (0.81), and relatively low means of out-degree (1.35) and betweenness centrality (1673.88). Before the pandemic began, these netizens would use Weibo infrequently, but due to the health crisis, they had become temporarily involved in Weibo topics about asking for help. Their online behaviors focus on pointing out problems, asking for information, seeking help, and providing support. This group is not keen on online discussion, and instead are more interested in following the issues they care about. They rarely attend online gatherings, and go offline once their goals are reached (see Figure 2B).

Cluster 3 comprises Shanghai netizens who are eager to participate in online relief. This group is more active on Weibo, though their number of followers (90,439.02) and posts (1782.62) are roughly equivalent to the average across all seven groups, their values for means of in-degree (62.10) and out-degree (15.58) are high. The Cluster 3 group has relatively high betweenness centrality (2,093,072.22) and closeness centrality (0.19), as seen in Table 5. Their online behavior primarily involves posting help-seeking information on Weibo and obtaining help and advice from other enthusiastic netizens. At the same time, they would also share their own experiences online to provide advice and support to netizens in similar situations. On Weibo, they form tight-knit, interactive groups around help-seeking topics (see Figure 2C).

Cluster 4 contains the volunteer organization accounts which emerged during the pandemic. As shown in Table 5, this group has high means of in-degree (25.33) and out-degree (89.24), as well as a high mean of betweenness centrality (8,393,435.28), although its number of followers (301.86) and posts (3435.19) are low in comparison to those of the other groups. Their online behavior is mainly reflected in actively searching for help-seekers on Weibo and using their own resources to provide them with help and support. For example, the Marine Compass Volunteer Team, one Cluster 4 account, had assigned staff in the Weibo super-topic square who were tasked to retrieve help-seeking information, match needs, connect channels, and recruit offline helpers. This type of node is displayed in the network graph in a radical shape (see Figure 2D).

Cluster 5 consists of Internet influencers and social media accounts of some renown. They possess a significant numbers of followers (9,250,003.31) and the highest number of posts (137,703.01) among the seven groups (see Table 5). However, their mean values of in-degree (7.52), out-degree (0.73), and closeness centrality (0.09) are low because they seldom respond to others’ mentions and comments. Ordinary users tend to ask them for help because of their online influence (see Figure 2E).

Cluster 6 constitutes famous media figures and influencers who are well-known at home and abroad. As shown in Table 5, this group has the highest number of followers (88,607,499.24) and a significant number of posts (61,781.43) of all seven groups. It has a relatively high mean of in-degree (62.86) but low means of out-degree (0.05), betweenness (0.00), and closeness centrality (0.00). Such accounts are used primarily to release information to the public, and rarely respond to users’ comments or mentions during the pandemic. These accounts have strong influences both online and offline, including organizations such as CCTV News and People’s Daily. During the pandemic, netizens tended to share information with such accounts and turned to their channels to highlight topics and try to draw the attention of the relevant aid departments (see Figure 2F).

Cluster 7 is composed of ordinary Internet users participating in online discussions. As shown in Table 5, they have a small number of followers (40,150.65) and posts (1820.83), but are very active in fueling discussions around online rescue topics. The Cluster 7 group has relatively low mean values of in-degree (1.07), out-degree (1.50), betweenness centrality (21,851.43), and closeness centrality (0.13). Their behavior resembles that of Cluster 2, as these netizens all participate in discussions out of their concern regarding the situation (see Figure 2G).

Outlier 1 is the Shanghai Post, the official Weibo account of the Information Office of Shanghai Municipality. With more than 9 million followers (mean of followers = 9,755,035.00), it enjoys national influence and is a critical window for the Shanghai Municipal Government to publicize as well as describe Shanghai’s economic and social development situations. It has the highest mean of in-degree (2043.00) and a relatively high number of posts (90,259.00), but the lowest means of out-degree (0.00), betweenness centrality (0.00), and closeness centrality (0.00). During the epidemic, many people sent information to Shanghai Post (using the @ function of Weibo) to express their demands, expecting their suggestions to be heard by the Shanghai Municipal Government (see Figure 2H).

Outlier 2 is the Weibo super-topic community, a chat forum developed by Weibo. As shown in Table 5, it has the highest number of followers (223,710,039.00) and the second-highest mean of betweenness centrality (21089535.53). However, it has relatively low values in the other indicators (i.e., mean of in-degree = 11.00, mean of out-degree = 423.00, and mean of closeness centrality = 0.20). During the lockdown period, to better gauge user opinions and respond to the demands of the masses, the Sina company issued a notice to users concerned about specific issues, announcing the launch of a “super-topic community” via a special account. Sina had set up an online community called “Shanghai Epidemic Help” to facilitate public discussion and, through a bot account, send targeted messages to accounts whose hashtags contained the keywords “Shanghai Epidemic Help” to invite them to participate in the discussion. The emergence of the bot account greatly improved the efficiency of information transmission (see Figure 2I).

Outlier 3 is Gangbiyangzi, a well-known local Shanghai blogger who has been active on the Internet during the lockdown period. As seen in Table 5, he enjoys the highest means of betweenness centrality (66,988,555.71), in-degree (315.00), and closeness centrality (0.22), and the second highest out-degree (158.00). He focuses on local affairs and holds some influence in Shanghai. During the pandemic, the account actively participated in discussions regarding epidemic prevention and mutual assistance among residents, continuously relaying information on patient assistance to the relevant authorities via his own account so that the voice of the people would be heard and responded to (see Figure 2J).

#### 4.1.2. The Structures of Online Self-Organized Communities

Community is one of the essential components of online self-organization. Community clustering aims to explore the patterns and structural characteristics of online self-organization. We explored the seven possible communities through the statistical inference in Section 3.1 and obtained 18 indicators for each community, which included the node number, the number of nodes from different self-organized groups, the community graph density, the community clustering coefficient, the average path length of the community, and the number of node behavior types in each community. Next, we used the 18 indicators to conduct K-means clustering for each possible community. The node number of each community represents the size of that community, and the number of nodes from different self-organized groups calculated the number of nodes coming from each of the seven groups as defined in Section 4.1.1. The community graph density refers to the degree of connectivity between nodes within a community. The community clustering coefficient represents the degree of interconnection between neighborhood nodes, and the larger the clustering coefficient, the more obvious the small group phenomenon is in the community. The average path length represents the shortest distance between each node and its farthest node in the community. The number of node behavior types in each community was calculated using the number of node behaviors related to offering help or advice, encouragement and support, other, or no response. The K parameter of community clustering was determined using the elbow method. In the end, the rescue self-organized community was divided into six patterns. The results are shown in Table 6.

**Figure 3:**
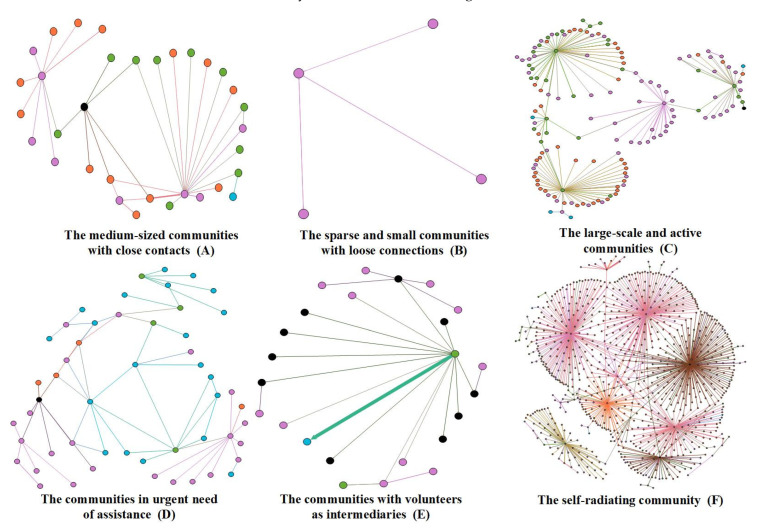
Patterns of Online Self-organized Communities.

Based on the clustering results with consideration to the actual current situation, the online rescue self-organized communities were divided into five patterns and one outlier. This section will analyze the characteristics and functions of each of these patterns (see Figure 3).

Cluster 1 refers to medium-sized communities with close contacts. As shown in Table 6, all mean values of members, number of nodes from different groups, and number of different behaviors within this pattern are in the middle range among those of all clusters. The mean values of the community clustering coefficients are the largest among all the clusters, indicating that the communities have a noticeable effect in terms of small groups and frequent interaction between nodes. In examining the information posted by these community members, we found that this community tends to discuss particular epidemic-related issues, such as the situation of the mobile cabin hospital, supply distribution channels, and the quarantine site environment. Most of these community members are stakeholders in the target topic, and are keen to discuss it to exchange information, to better understand the situation, and to find emotional release. Most of these participants are ordinary netizens who are very much affected by the pandemic, who engage in the discussions temporarily. The community topics develop continuously, and constantly expanding thanks to new information shared by a few community activists, and new rounds of discussion are continuously raised. The characteristics graph of this pattern is shown in Figure 3A.

Cluster 2 represents the sparse or small communities with loose connections. As seen in Table 6, their membership is small and consists mainly of ordinary Internet users. The community in this pattern have infrequent internal discussions and limited influence online. Information disseminated by community members mainly involves online help, situation reports, and advertising. Community members are not motivated to exchange views, will often disappear from the Internet after posting information or achieving their desired purpose, and rarely participate in follow-up questions or discussions. Due to the lack of participation or engagement, this community’s size is the smallest among the clusters. The characteristics graph of this community is shown in Figure 3B.

Cluster 3 consists of large-scale and active communities, with the highest number of members across the five patterns. As shown in Table 6, this pattern includes a number of activists, such as members from Cluster 1 and Cluster 3 as defined in Section 4.1.1, who effectively fuel information dissemination and discussion within the community, leading to the increased community size. A review of the information released by the community revealed that the community concentrates on timely issues that need to be addressed, such as living conditions in quarantine sites, community services, procurement of supplies, and mobile cabin hospital environments. There is a phenomenon of empathy in the community, where its members tend to gripe and complain about a particular issue, which then leads to more discussion and, ultimately, an emotional consensus within the community. The characteristics of this community are shown in Figure 3C.

Cluster 4 consists of the communities in urgent need of assistance. Communities in this pattern organize themselves primarily around emergency services, with topics of posts including emergency surgery or positive nucleic acid tests. Members of this community flooded to the accounts of Cluster 1, Cluster 5, and Cluster 6 as defined in Section 4.1.1 with messages sent via the Weibo @ function to draw the attention of celebrities and key media so that problems might be fixed quickly (see Table 6). These community members disseminate information with a strong purpose, wanting help-seekers to know they are cared for and to help them pull through their difficulties quickly. As a result, there is less communication within the community, and the information transmission is manifested as suggestions provided directly or help to deliver information to important network nodes, which explains the higher ratio of the mean value of non-responsive nodes to the mean value of community members. The characteristics of this community network are plotted in Figure 3D.

Cluster 5 comprises communities with volunteers acting as intermediaries. As seen in Table 6, the mean values of members, number of nodes from different groups, number of different behaviors, and clustering coefficient are all under the midpoint. The main characteristic of this community is that it is involved with networking between members through volunteers. For example, the accounts of Shanghai Defender, a volunteer team, posted to seek out netizens in need through several of Weibo’s functions (i.e., post search, super-topics recommendations), as well as leaving messages in the help-seekers’ comment areas to provide them with assistance and contact information. The community in this pattern is not limited to a specific topic or post, but rather spans a wide range of information topics regarding assistance. As long as netizens need help, a community may be formed in this pattern through the center node of a volunteer account. The characteristics graph of this community is shown in Figure 3E.

Outlier 1 represents the self-radiating community. There are several self-radiating netizens who are both help-seekers and information sharers. A community grew out of their signals for help, and continues to expand through their constant updates. In the process, many zealous users join in to offer them suggestions or encouragement, which in turn incentivizes the original poster to share more information. At the same time, their experience in seeking assistance sets an example for others in similar predicaments. Hence, the repeated interactions between help-seekers and providers creates multiple information cascades, significantly enhancing the community’s activity. The characteristics of this community network are shown in Figure 3F.

#### 4.1.3. The Evolution of Online Self-Organizations

**Figure 4:**
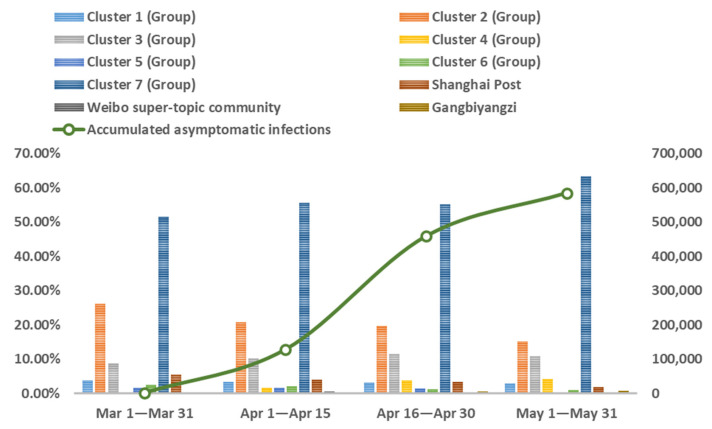
The Distribution of Self-Organized Groups in the Different Stages of Lockdown.

**Figure 5:**
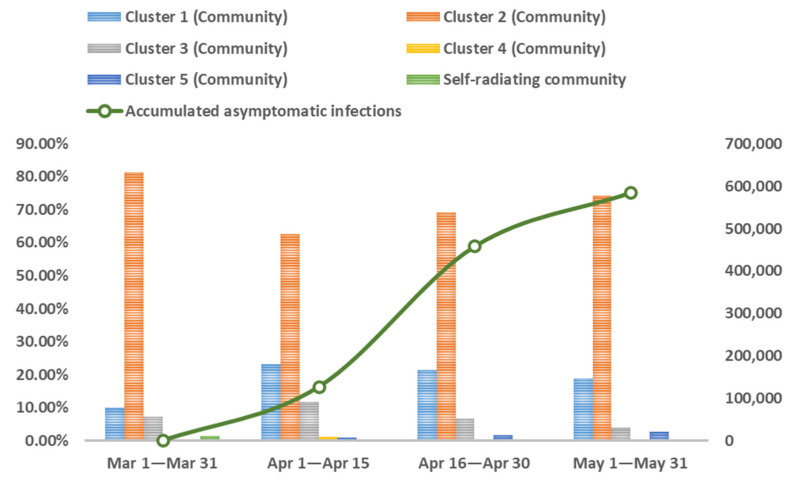
The Distribution of Self-Organized Communities in Different Stages of Lockdown.

**Figure 6:**
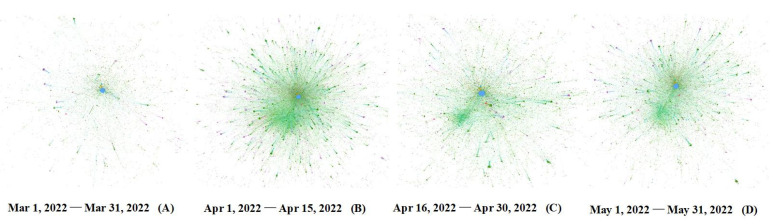
The Evolution of the Networks of Online Self-Organizations.

Analyzing the evolution of online self-organization based on the patterns of self-organized groups and communities can amplify the microscopic process of self-organized rescue during public health emergencies. The cumulative trend graph of the daily new asymptomatic cases in Shanghai during the defined period shows that the shape of the cumulative growth curve of asymptomatic infections conforms to the logistic growth model. Therefore, according to its characteristics, the process of online self-organization evolution can be divided into four stages. We calculated the distribution of the different patterns of online self-organized groups and communities at different stages and mapped the evolutionary network. The results are shown in Figure 4, Figure 5 and Figure 6.

As seen in Figure 4 and Figure 5, at each stage of the pandemic, the self-organized relief groups were composed primarily of ordinary netizens and ad hoc participants, and the community pattern was also based on the sparse and small communities formed by them. Specifically, the first stage (1 March 2022 to 31 March 2022) was the initial phase of lockdown when there were relatively few asymptomatic infections. The proportion of medium- and large-scale communities was relatively small, and the network of online self-organization was scattered, as shown in Figure 6A. In the second stage (1 April 2022 to 15 April 2022), the number of asymptomatic infections exploded, and the obstacles caused by the epidemic and lockdown were fully revealed. To solve practical problems in real life, Internet users actively participating in self-organized rescue began to increase significantly, and volunteer groups emerged. Common issues such as panic buying of goods, supply distribution problems, and effects of centralized isolation began to arouse wide-spread attention and discussion, leading to an increased proportion of medium- and large-scale communities. At this stage, the densest online self-organization is reflected through active online activities, as shown in Figure 6B. The third stage (16 April 2022 to 30 April 2022) witnessed the resolution of the problems that emerged in the previous stage, and volunteer groups became skilled in their rescue activities. When comparing the second and third stages, the proportion of self-organized groups did not alter significantly. Figure 6C shows a decline in the proportion of large-scale communities regarding certain specific issues as well as of communities in urgent need of assistance, as well as a slow decline in the density of the overall network. In the fourth stage (1 May 2022 to 31 May 2022), with the pandemic now under control, the number of self-organized rescue groups decreased significantly, and medium- and large-scale communities established to address some of the previously common problems declined significantly. Network activity also decreased, resulting in sparser network density, as shown in Figure 6D.

### 4.2. The Rescue Mechanism of Online Self-Organizations in Public Emergencies

As mentioned above, online rescue self-organizations effectively deal with intense, large-scale special situations that arise out of public health emergencies, partly compensating for the emergency needs triggered by environmental uncertainties and information asymmetries during the crisis [28]. The fragmented and scattered urgent emergency problems cannot wait for a unified response handled centrally, and instead are solved in one-to-one or many-to-one manners [4]. In this section, we examined the rescue mechanism of online self-organizations amidst public health emergencies, according to relevant materials and the research findings shared in Section 4.

**Figure 7:**
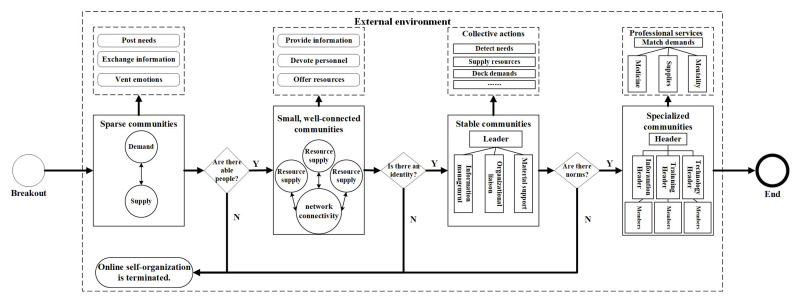
The Mechanism of Online Self-Organized Rescue.

The rescue mechanism of online self-organizations during public emergencies is shown to contain four core parts (see Figure 7).

(1) Groups Gathering Based on Common Purposes

In the early days of traditional offline self-organization, mobilization of the masses for collective action relied on the will, interest, and relationship networks of able citizens [29]. However, during public health emergencies, the rapidly changing environment and information vacuum leads to able citizens—ordinary people—seeking information to understand the crisis development trend and to ensure their own safety. Therefore, online self-organized rescues originate from groups gathering based on their common purposes, through posted requests, information exchange, and emotional expression. Through this process, many loosely-connected communities spring up online, some of which dissipate after achieving their goals while others evolve into small, well-connected communities with frequent information exchange as their members share similar experiences and common awareness.

(2) Loose Groups Formed by Core Members

During a public health emergency, two types of core members play a crucial role in the maintenance and development of the group: those responsible for resource supply and those with network connectivity. Those responsible for resource supply build group trust and realize rescue efforts by devoting their resources towards it [18]. For example, members with information resources continuously released first-hand information to ensure the frontline situation was understood, while those with specific skills dedicated their knowledge and strength to the group and helped solve professional problems. Meanwhile, the members with network connections may work to curb demand and allocate resources [47]. For example, community members with professional connections to drug providers and couriers helped coordinate or track deliveries to address requests for chronic disease medications. Both types of people were extremely capable. The former was more common in online mutual rescue groups, and when the problem was solved or resources exhausted, they would fade off the Internet. The latter served as an important basis for group maintenance, as they enjoyed a certain amount of social status and prestige, and were adept at persuasion and mobilization, advancing resource allocation through their relationship networks. In some cases, groups’ core members featured both resource-providing and network-connecting individuals.

(3) Stable Organizations According to Their Sense of Identity

During public health emergencies, driven by altruism, group members complete initial rescues through collective actions, shaping the early organizational principles [48].

Through this process, members establish trust and develop a consensus and a sense of identity [49,50]. More importantly, the group’s abilities in management, coordination, and implementation are tested fully. Successful rescues help enhance organizational cohesion and generate a strong sense of acquisition and self-confidence. Through the mobilization of core members, clearer organizational goals emerge, identity is reinforced, and organizational activities continue to develop, resulting in a relatively stable organizational structure, explicit internal divisions of labor, and precise work procedures.

(4) Specialized Organizations Based on Norms and Standards

With the development of the organization and the deepening of its internal identity, outsiders with similar values or ideas are increasingly absorbed into the organization, leading to the expansion of the scale of the organization and branching in its structure. To ensure the continuation of collective activities, the rough rules begin to become standardized and institutionalized, and evolve to be integrated into the organizational code of conduct [51], implying that the organization has gradually became formal and professional. During this process, the organization transitions from being entirely open to becoming relatively closed, indicating that new participants in the community must follow an enrollment procedure during which they agree with the organizational philosophies and norms [52,53].

## 5. Discussion

Previous research has largely illustrated the role of social media as informational support during disaster rescue activities [13,18], while the functioning of online self-organizations has been largely ignored by existing literature. This study aimed to address this gap in knowledge in three ways: (1) identify the compositions and patterns of online self-organized groups; (2) reveal the main constituent of online self-organized communities; and (3) explore the rescue mechanism of online self-organizations during public emergencies. Our findings are significant because, with the increasing popularity of online self-organizations in relief activities, understanding the functioning of online self-organizations can help guide the authorities in formulating suitable policies to incubate and manage these organizations so that online self-organizations can form quickly to respond to citizens’ needs during emergencies.

### 5.1. Theoretical Implications

This article used big data analysis methods such as machine learning and social network analysis to comprehensively explain the online self-organization process during public health emergencies, aiming to address the lack of previous research into these high-frequency interactive relationships, and to reveal some of the unique characteristics of online self-organizations.

First, we used the BERT model to classify the online response behavior of network users, and combined the results with user attribute data and social network indicators to cluster the network users participating in online self-organization. Ultimately, we were able to determine seven types of users participating in online self-organizations during public health emergencies. We found that the composition of online self-organization participants follows the Pareto principle, which reflects the findings of Silver and Matthews (2015) who found that the majority of participants in online self-organizations provide only basic help and then disappear from the network [31]. Additionally, during the clustering process, the research team also found that social robots also participated in online self-organization rescue activities, which significantly improved the efficiency of search and rescue efforts as well as the expanse of information dissemination. Tu (2022) has also noted that bot accounts can improve information discovery [3].

Second, we studied the formation and clustering of online communities during a public health emergency (specifically, the COVID-19 lockdown period in Shanghai, China) and found that small communities made up 70.96% of the total number of communities. These small communities had simple connection methods and were short-lived, usually dissolving after achieving their goals. Through community clustering, the research group also found a unique type of community called the “self-broadcast communities” which is made up of users who have personal experience related to the event. They share their experiences and feelings in real-time online, allowing for interaction and discussion with the public to alleviate confusion and information asymmetry caused by the public health emergency. As suggested by Hughes and Tapia (2015), activists within digital voluntary groups were those who most actively searched for help information [14].

Third, we analyzed the evolution of online self-organizing networks during a public health emergency and found that the level of activity in online communities was strongly correlated with the severity of the outbreak. Specifically, as the outbreak became more severe, more and more diverse self-organized communities formed online. Conversely, as the outbreak lessened, fewer communities formed. The research group also summarized and concluded that the mechanism of online self-organization during a public health emergency is different from that of traditional self-organization contexts [2,26]. The research group found that online self-organization during the early stage of a public health emergency relies more on a common goal rather than on individuals who can mobilize others. Specifically, we identified four core stages of online self-organization: initial aggregation of the group, formation of core groups, collective action, and establishment of organizational norms. These four stages are crucial for the sustained development of online self-organization. This is in line with the previous finding that self-organizations build themselves from disordered to well-organized [5].

### 5.2. Practical Implications

This study provides practical implications for the management of online self-organizations in responding to crises. First, online self-organizations have assumed considerable importance in public health emergencies [3,5,24]. Online self-organizations are built to meet citizens’ numerous needs and are able to take advantage of informational support offered by social media to respond to goals that formal, official organizations are unable to fulfill. When a crisis occurs, the relevant departments should actively build an online communication platform for those affected, such as discussion forums for specific issues or special help-seeking groups, to accelerate information-gathering and promote the formation of self-organizations.

Second, for professional voluntary organizations emerging in the online rescue process, social media can be used to establish an authentication mechanism and empower participants through intelligent information technology [54]. For example, for certified voluntary teams, social media platforms can provide them with social bot accounts, group communication systems, document processing systems, and procedure planning systems, all for free, thereby helping them to improve their productivity.

Third, authorities should encourage professional media practitioners to conduct online interactive live streams about the issues most pressing or concerning to the public during the crisis [55]. This will help eliminate the information fog, dismissing incorrect information and reducing the development of rumors. For example, during Shanghai’s lockdown, many people with asymptomatic infections were very concerned about the conditions of the mobile cabin hospitals. However, patients being received in the mobile cabin hospitals earlier on were able to share information about the treatment process to others outside, which effectively dispelled doubts of outsiders and the wider public and enhanced citizen cooperation with the government.

Finally, self-organizations are not a panacea for dealing with all types of issues associated with public health emergencies, and are sometimes incompetent at coping with time-sensitive or highly professional rescue tasks that require full authorization [56]. Therefore, it is recommended that government departments set up special teams to manage such tasks. Additionally, as they can be at risk of being influenced by capable people and core groups within the organization, sometimes self-organization goals and identified values may deviate from those of the government or from wider social values. In this case, relevant departments should intervene in a timely manner to address such deviations and avoid losing control of situations due to such self-organizations.

### 5.3. Limitations

Although this study does shed light on the organizational development of online self-organizations, there are nonetheless some limitations to this study. First, Weibo users do not fully represent all Chinese netizens. Only 40.9% of Chinese netizens use Weibo, and the proportion of users aged 20 to 50 is just under 60%, which means that this study is more applicable for this specific demographic. Second, not all online self-organized rescue activities are carried out through Weibo. However, our research focused only on those generated through Weibo. Third, the research materials came mainly from public information and data generated during the online self-organized relief activities, including help-seeking and rescue information, unclassified documents, and Weibo users’ interactions, so some processes that cannot be recorded may have been overlooked in our study. Future research could try to use inference methods to predict users’ demographic information, and combine the data and functions offered by other types of online social media platforms to improve the limitations of the existing research.

## 6. Conclusions

This study profiled the functioning of online self-organizations from the perspectives of patterns and mechanism. Combining data mining techniques (i.e., BERT and K-means clustering) and content analysis, we traced netizens’ behaviors during the COVID-19 lockdown period in Shanghai to classify the patterns of online self-organized groups and communities. Our analyses showed that the self-organized groups can be divided into seven patterns, and only certain key groups truly participated in the rescue activities. The self-organized communities comprised five patterns, among which sparse and small communities dominated task resolving. The evolution of online self-organizations was shown to comply with the logistic growth model. By analyzing public documents and website information, we identified the mechanism of online self-organizations as involving four core parts. Our research provides a new insight into the integration of social media and online self-organized rescue activities. The patterns and mechanism can serve as references for the policymakers when formulating policy support for the formation and development of online self-organizations in the future so that more needs can be met more quickly during public health emergencies.

## Figures and Tables

**Table 1:** The Values of Social Media in Crisis Management.

Values	Examples
Discovering information to evaluate crisis situations.	De Albuquerque et al. (2015) used Twitter data during floods in Europe to assess the severity of disasters [38].
	Huang and Xiao (2015) constructed a classification system for information based on Twitter data to help aid workers estimate the different stages of crisis evolution [18].
	Kryvasheyeu et al. (2016) analyzed Twitter data before, during, and after Hurricane Sandy and found that disaster damage was highly correlated with Twitter information exchange [19].
Matching individuals’ needs through aid offers and requests.	Pourebrahim et al. (2019) found that during Hurricane Sandy, many victims used social media to obtain weather information due to power outages [13].
	Li et al. (2019) showed that in the aftermath of a crisis people used social media not only for disaster information, but also for emotional support [39].
	Zou et al. (2019) found that widespread damage caused by Hurricane Harvey overloaded the 911 system, leading many disaster victims to ask for help on social media [40].
	Wang et al. (2021) found that the more frequently community residents used Twitter, the more relief they received [20].

**Table 2:** Classification of Netizens’ Replies on Weibo.

Types of Replies	Annotated Amount	Examples
Help and advice	2436	Contact the community, and tell the community to contact the epidemic prevention center. Call at any time, don‘t waste time, and ask more places and there will always be a solution.
Encouragement and support	2039	Hold on, People of Shanghai!
Other	2525	Express packages are piling up and cannot be delivered in the Pudong Area.

*Note:* Example replies are translated from the original Chinese posts.

**Table 3:** Accuracy Rate, Recall Rate, and *F*-Value of BERT Model.

Types	Accuracy Rate	Recall Rate	*F*-Value
Help and advice	0.8288	0.8124	0.8205
Encouragement and support	0.9837	0.9717	0.9777
Other	0.9231	0.9423	0.9326

**Table 4:** Number of Netizen Replies.

Types	Number	Percentage
Help and advice	13,430	11.93%
Encouragement and support	13,721	12.19%
Other	55,376	49.21%
No response	30,001	26.66%

**Table 5:** Clustering Results of Online Self-Organized Groups.

		Cluster 1	Cluster 2	Cluster 3	Cluster 4	Cluster 5	Cluster 6	Cluster 7	Outlier 1	Outlier 2	Outlier 3
Mean of followers		1,622,873.46	9811.44	90,439.02	301.86	9,250,003.31	88,607,499.24	40,150.65	9,755,035.00	223,710,039.00	961,230.00
Mean of posts		43,643.85	1782.62	4189.75	3435.19	137,703.01	61,781.43	1820.83	90,259.00	14,861.00	50,633.00
Mean of in-degree		2.20	0.81	62.10	25.33	7.52	62.86	1.07	2043.00	11.00	315.00
Mean of out-degree		1.22	1.35	15.58	89.24	0.73	0.05	1.50	0.00	423.00	158.00
Mean of betweenness centrality		29,038.48	1673.88	2,093,072.22	8,393,435.28	31,815.66	0.00	21,851.43	0.00	21,089,535.53	66,988,555.71
Mean of closeness centrality		0.19	0.81	0.19	0.19	0.09	0.02	0.13	0.00	0.20	0.22
Types of replies	Help and advice	171	2727	205	21	8	0	5768	0	0	1
	Encouragement and support	127	2501	55	0	8	0	5054	0	0	0
	Other	383	9095	98	0	21	1	18,529	0	1	0
	No response	395	15	10	0	116	20	1842	1	0	0
Number of accounts		1076	14,338	368	21	153	21	31,193	1	1	1

**Table 6:** Clustering Results of Online Self-Organized Communities.

	Cluster 1	Cluster 2	Cluster 3	Cluster 4	Cluster 5	Outlier 1
Mean of community members	40.498	3.976	154.442	57.889	24.476	840.000
Mean of group numbers of netizens active on Weibo	0.916	0.080	3.023	7.333	0.524	3.000
Mean of group numbers of netizens who temporarily participate in discussions	12.093	2.389	33.085	17.667	3.429	353.000
Mean of group numbers of netizens eager to participate in online relief	0.366	0.030	1.124	0.389	0.095	6.000
Mean of group numbers of volunteer accounts emerging due to the pandemic	0.000	0.000	0.000	0.000	1.000	0.000
Mean of group numbers of Internet influencers and media accounts of some renown	0.000	0.000	0.000	1.000	0.000	0.000
Mean of group numbers of famous media and influencers well-known at home and abroad	0.002	0.003	0.008	0.778	0.000	0.000
Mean of group numbers of ordinary Internet users participating in online discussions	27.007	1.446	116.946	30.389	19.333	476.000
Ratio of the mean of help and advice nodes to the mean of community members	22.88%	19.77%	15.75%	16.70%	18.68%	11.79%
Ratio of the mean of encouragement and support nodes to the mean of community members	18.88%	14.16%	13.83%	9.31%	4.47%	65.36%
Ratio of the mean of other nodes to the mean of community members	53.41%	59.31%	67.33%	35.32%	61.48%	22.38%
Ratio of the mean of no response nodes to the mean of community members	4.83%	6.76%	3.09%	38.67%	15.37%	0.047%
Mean of community graph density	0.056	0.450	0.009	0.027	0.117	0.001
Mean of community clustering coefficient	0.042	0.000	0.030	0.033	0.015	0.014
Mean of average path length	1.944	1.080	2.449	1.617	1.356	2.724
Number of communities	440	1488	129	18	21	1

## Data Availability

The data sets supporting the conclusions of this article are available from the corresponding author upon request.
